# Pyrolysis behavior of *Sterculia guttata* shell biomass: kinetics, thermodynamics, techno-economic and life cycle assessment of industrial-scale biochar production

**DOI:** 10.1039/d5ra09614f

**Published:** 2026-02-23

**Authors:** Subramaniyasharma Sivaraman, Rangabhashiyam Selvasembian

**Affiliations:** a Department of Environmental Science and Engineering, School of Engineering and Sciences, SRM University-AP Amaravati Andhra Pradesh 522240 India rambhashiyam@gmail.com; b Centre for Interdisciplinary Research, SRM University-AP Amaravati Andhra Pradesh 522240 India

## Abstract

To address the growing demand for renewable energy sources in developing economies, this study evaluates the pyrolysis behavior, kinetics, and thermodynamic characteristics of *Sterculia guttata* shell waste (SGSW) to determine its suitability as a bioenergy feedstock. Kinetic parameters were calculated using model-free isoconversional techniques. The average activation energies (*E*_a_) acquired varied between 50.21–71.86 kJ mol^−1^, suggesting moderate energy needs for pyrolysis. Reflecting the complicated response mechanism, the pre-exponential factor (*A*) varied from 10^6^ to 10^7^ min^−1^. Thermodynamic study produced enthalpy changes (Δ*H*) between 22.9 and 54.8 kJ mol^−1^ and Gibbs free energy (Δ*G*) values of 152.1 to 156.8 kJ mol^−1^, demonstrating the endothermic and feasible nature of the process. The study further establishes industrial viability through a techno-economic analysis, which projects a Return on Investment (ROI) of 38.69%, a payback period of 2.7 years, and a Net Present Value (NPV) of USD 6.67 million. The life cycle assessment results underscore that SGSW biochar sustainability is sensitive to allocation methods, with GWP values ranging from 0.88 to 1.27 kg CO_2_ eq per kg of biochar. These findings underscore the scientific value of SGSW by validating it as a low-energy, economically robust candidate for scalable thermochemical conversion and sustainable bioenergy production.

## Abstract 2

This study evaluates *Sterculia guttata* shell waste (SGSW) as a sustainable bioenergy feedstock using pyrolysis kinetics and thermodynamic analysis. Techno-economic and life cycle analyses support viability and sustainability at scale.

## Graphical abstract

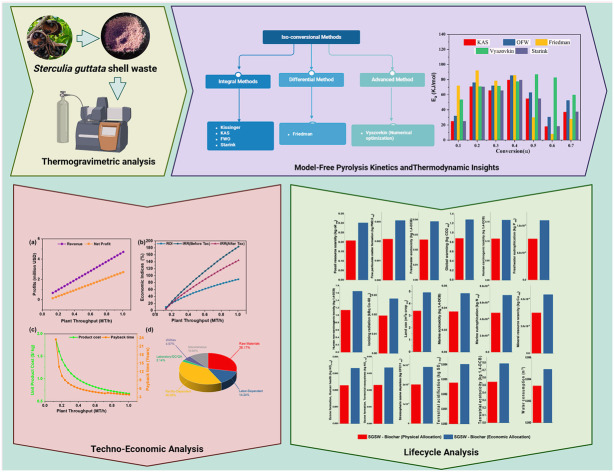

## 1 Introduction

The global energy demand is met primarily by oil (30%), natural gas (23%), coal (27%), nuclear (5%), renewable energy sources (12%), and biomass (3%).^1^ The current output from renewable sources is still relatively small in comparison to that of non-renewable energy sources, but it holds considerable promise for enhancing fuel quality. The declining accessibility of fossil fuels and the ecological harm they cause have attracted worldwide attention towards renewable energy options, especially biomass, with a focus on lignocellulosic biomass. This biomass is acknowledged as a promising resource to tackle global energy challenges, given its worldwide demand.^2^ So efforts are being made to promote effective biochemical and thermochemical techniques for the global use of various new biomass sources.^3^ Increasing global concerns about climate change have aligned with an intensified emphasis on global greenhouse gas emissions. Focusing on renewable and sustainable energy sources is guiding investigations into the integration of biomass-derived carbon within the various industrial processes.^4,5^ Pyrolysis is a conversion technique that changes biomass into solid, liquid, and gaseous products.^6^ The analysis of the thermal breakdown of coal and biomass is essential for assessing their applicability and for improving the pyrolysis process in anticipation of scaling up.^7,8^ The recycling of waste holds considerable importance, not just for effective waste management and energy recovery, but also for improving the quality of materials.^9^

*S. guttata*, referred to as “wild almond”, is a medium to large deciduous tree indigenous to the Indian subcontinent and certain regions of Southeast Asia, encompassing India, Bangladesh, the Andaman and Nicobar Islands, Myanmar, Thailand, and Laos.^10^ In India, it is prevalent in several places, including Maharashtra, Karnataka, Kerala, Tamil Nadu, Assam, and Gujarat, often thriving in the wild along riverbanks, forest edges, and occasionally in gardens or urban settings. The tree may grow to heights of up to 15 meters and is noted for its unique woody fruit capsules and edible seeds.^11^ A single mature *Sterculia* sp. A tree can produce approximately 350 kg to 2000 kg of seeds annually.^12,13^ Tribal communities utilize the seeds for food source and other value-added applications.^14,15^ Following seed extraction, its shell (44% wt.) is typically considered waste.^16,17^ This renders SGSW a prospective contender, akin to other biomass residues utilized for renewable energy. Thermogravimetric analysis is the predominant method employed to assess thermal degradation and conduct kinetic investigations.^18,19^ The change from a linear fossil-based economy to a circular bio-economy has made the thermochemical conversion of lignocellulosic waste very important. Pyrolysis is the most interesting of these conversion routes because it may turn waste biomass into high-value energy products including bio-oil, pyrogas, and solid biochar. Biochar has been used for more than only improving soil in the last several years.^20^ Recent studies have shown the adaptability of biomass derived biochar in sophisticated environmental and applications such as wastewater treatment,^21,22^phytoremediation^23^ and catalysis.^24,25^ To engineer biochar with the specific physicochemical properties required for these advanced applications and to maximize energy yields precise control over the thermal degradation process is non-negotiable. This necessitates a profound understanding of thermal degradation kinetics. Kinetic parameters (activation energy, pre-exponential factors) serve as the mathematical backbone for predicting biomass behavior under thermal stress. A significant body of literature has examined the pyrolytic behavior of various feedstocks to derive these parameters.^26–28^ They examined the pyrolytic behavior of biomass and derived kinetic parameters based on which the thermodynamic parameters were established. In this study, we utilize various pyrolysis kinetic models, including Flynn–Wall–Ozawa (FWO),^29^ Friedman,^30^ Kissinger–Akahira–Sunose (KAS),^31^ Starink,^32^ and Vyazovkin^33^ methods, which were employed to estimate the behavior of SGSW biomass during pyrolysis. These models facilitate the computation of kinetic characteristics, such as activation energy, which assist in predicting and understanding thermal degradation processes.^34,35^ The development and optimization of pyrolysis operations rely on the understanding of kinetic parameters.^36,37^ This information directs the choice of operational parameters, including temperature and heating rate, to guarantee efficient and economical energy production from biomass.^38,39^ Alongside kinetic parameters, thermodynamic data (Δ*H*, Δ*S*, Δ*G*) are important for assessing process feasibility, energy demand, and spontaneity, guiding energy integration strategies for scale-up.^40^ It enables researchers to ascertain the optimal conditions for biomass degradation, thereby yielding the necessary energy products, including biochar, bio-oil, and pyrogas.^39,41^ Only optimization of reaction chemistry is insufficient for industrial adoption. A major barrier to the commercialization of biomass pyrolysis technologies is the lack of comprehensive Techno-Economic Analysis (TEA).^42,43^ Without a rigorous assessment of process feasibility, capital costs, and return on investment, the transition from laboratory scale to industrial scale remains sluggish.^44,45^ A study conducted by Parmar *et al.*^46^ showed a comprehensive analysis of the combined effects of reaction kinetics and heat transfer on the pyrolysis of sugarcane bagasse biomass and found that the scalability of biomass pyrolysis is based on the constituent components. The lack of comprehensive TEA is a major barrier to the commercialization and integration of biomass pyrolysis technologies.^45,47,48^ Without understanding the techno-economic performance, up-scaling and market adoption remain slow.^49,50^ Zepeda *et al.*^51^ present a TEA and sensitivity analysis for a biochar-based slurry fuel plant processing rice straw. Integrating biomass kinetic studies with TEA is a recent strategy that can better explain the efficiency and feasibility of biomass pyrolysis systems, supporting future commercialization and investment decisions. Ramesh and Somasundaram^52^ have performed a comprehensive investigation of *Parthenium hysterophorus* pyrolysis, uniquely integrating kinetic modeling and TEA. Sasidhar *et al.*^53^ utilized iso-conversional kinetic modeling and response surface methodology to investigate the thermal degradation of milled coffee wastes, with TEA confirming the process's financial viability (14% IRR, 9.18-month payback). These works detail exploiting the latent energy potential in byproducts to convert them into valuable resources, hence alleviating the harmful environmental effects associated with the traditional incineration method, yet significant knowledge gaps persist for novel, uncharacterized feedstocks. Its industrial deployment hinges on the ability to accurately predict product yields and compositions and to assess economic feasibility and environmental sustainability. Integrating these domains is essential for process optimization, scale-up, and sustainable energy production, yet presents significant methodological and practical challenges. This report synthesizes recent advances, methodological frameworks, and research gaps in the combined application of pyrolysis kinetics and TEA and LCA, with a focus on bridging theory and practice for industrial and sustainability outcomes. This study presents the first comprehensive investigation into the pyrolysis of SGSW. To address these gaps, this study presents the first comprehensive investigation into the pyrolysis of SGSW. The primary contribution of this work is the synergistic integration of robust kinetic modeling with techno-economic analysis. Specifically, we employ a suite of five iso-conversional models (FWO, KAS, Friedman, Starink, Vyazovkin) to determine the fundamental activation energies and thermodynamic parameters governing SGSW degradation. Crucially, we move beyond thermochemical characterization by using this data to ground a rigorous TEA and LCA, thereby establishing the essential baseline for process feasibility, energy integration, and commercial viability. This integrated approach aims to provide the foundational data required to transition SGSW from an agricultural waste liability into a sustainable resource for energy and advanced material applications.

## 2 Materials and methods

SGSW was collected, thoroughly cleaned to remove impurities, and oven-dried for 25 hours at 105 °C to remove moisture. The desiccated biomass was then ground and sifted to obtain a uniform particle size of less than 200 µm, ensuring consistency in subsequent analyses.^54^ Analytical grade reagents were used for all experimental procedures. Acetone-99%, hydrochloric acid-37% were purchased from Avra chemicals (India). DCM GC-HS grade was procured from SRL Chemicals (India). High-purity nitrogen gas (99.999%) employed as the inert carrier gas for both the thermogravimetric analysis and the fixed-bed pyrolysis reactor.

### 2.1 Biomass characterization

Proximate analysis was carried out on the biomass in accordance with the American Society for Testing and Materials (ASTM). The fixed carbon content of the biomass was estimated using the following eqn (1) (ASTM D7582-15, 2020):

1 Fixed carbon (%) = 100 − (volatile carbon (%) + Ash (%) + moisture (%))

where the moisture content (ASTM D3175-20, 2020), volatile matter (ASTM D3175-20, 2020), and fixed carbon and ash content (ASTM D3174-12, 2018) were determined using their respective ASTM procedure.^39^ An elemental analyzer (VARIO-EL CUBE, Elementar, Germany) was applied for quantitative analysis of CHNS elements. The composition of the bio-oil collected from each condition was characterized using Gas Chromatography-Mass Spectrometry (GC-MS) using a Shimadzu GC-MS System with a Single Quadrupole QP2020 Mass Spectrometer. The aqueous phase was extracted using acetone, and the extracted phase was diluted 100 times. The samples were injected into GC at an injection temperature of 230 °C, flow at 1 mL min^−1^, oven temperature of 50 °C, ion source at 230 °C, and interface at 280 °C with solvent cut time of 3 minutes.^55^ The compounds were identified using the NIST (National Institute of Standards and Technology) mass spectral library.^56^

### 2.2 Thermogravimetric analysis

The TG209 Libra thermogravimetric analyzer (NETZSCH, Germany) was utilized for the thermogravimetric analysis.^57^ An alumina crucible containing roughly 5 mg of the sample at rates of 5, 10, and 15 °C min^−1^ was heated from room temperature to 900 °C while being supplied at a flow rate of 20 mL per min nitrogen.^58^ The recorded TGA data was analyzed using the MixChar program,^59^ a tool designed to estimate the composition of lignocellulosic biomass based on thermogravimetric data. MixChar deconvolutes the TGA curve to approximate cellulose, lignin, and hemicellulose proportions in the biomass sample.

### 2.3 Pyrolysis kinetic modeling of *S. guttata* shell

A non-isothermal thermogravimetric analysis can employ several mathematical approaches to ascertain the devolatilization kinetics of biomass pyrolysis by TGA.^26,39^ The following eqn (2) can illustrate a process perspective that elucidates the thermal degradation of SGSW.^60^2
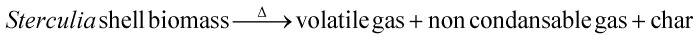


Eqn (3) delineates the relationship between time and mass conversion in biomass thermal degradation.^61^3
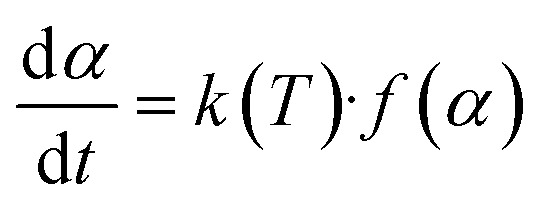
*f*(*α*) is reliant upon a particular conversion value *α*.

The rate constant *k*(*T*) is represented in the Arrhenius equation in the following manner.4
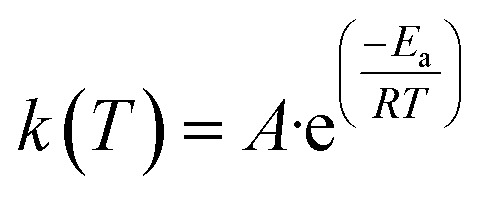
where *A* represents the pre-exponential factor (min^−1^), absolute temperature (K), *R* represents the gas constant (8.314 J mol^−1^ K^−1^) and *E*_a_ represents the apparent activation energy (kJ mol^−1^).^6^

When we combine eqn (3) and (4), we get the following expression for the conversion rate5
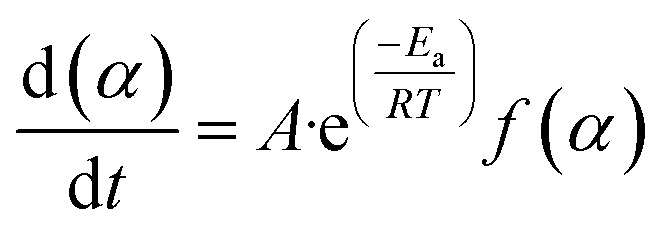
where the data of mass loss from the decomposition experiment are used to compute (*α*).^39^6
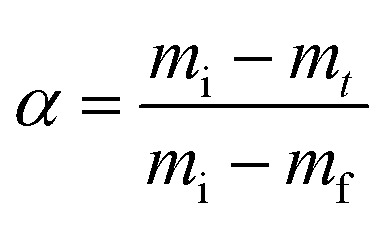
In the present setting, *m*_*t*_ (mg) indicates the biomass sample's mass at a specific time, *m*_i_ denotes the biomass' initial mass (mg), and *m*_f_ represents the biomass' final mass (mg).^62,63^

Eqn (5) and (6) are applicable in eqn (7) for the calculation of the conversion rate.7
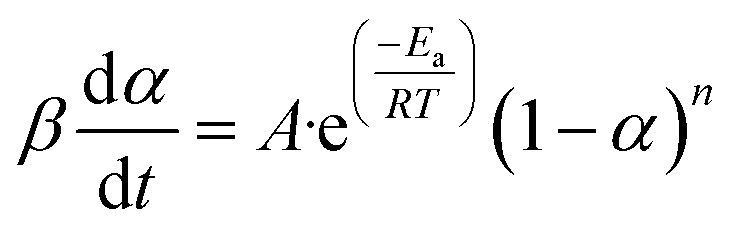


The first-order rate equation is expressed as follows (8) (ref. 64)

8 *f*(*α*) = (1 − *α*)^*n*^

Following the rearrangement and integration of eqn (9),9
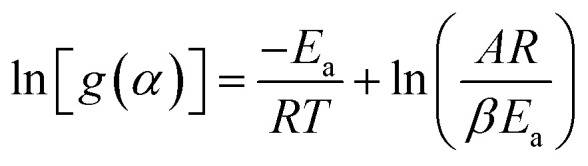


The TG/DTG data and the Arrhenius equation were utilized to ascertain the kinetic triplets, which comprise pre-exponential factor (*A*) and the activation energy (*E*_a_). Analytical solution of eqn (9) followed by a mathematical estimate for the exponential component can also be used to determine these kinetic parameters.^62,65^

### 2.4 Model-free isoconversional methods

The thermogravimetric data of *S. guttata* can be modeled using numerous approaches. Model-free and model-fitting are the two categories of non-isothermal kinetics.^66^ The iso-conversional method, or model-free method, is the predominant technique utilized in the kinetic analysis of biomass pyrolysis.^19^ Generally, integral and differential are two categories of model-free methods. The differential iso-conversional method's reliance on the instantaneous rate value makes it vulnerable to experimental noise, leading to instability in the numerical value.^67^ In the TGA experiment, while applying the integral method, it effectively mitigates this phenomenon. The Kissinger–Akahira–Sunose (KAS) integral method and the Flynn–Wall–Ozawa (FWO) integral method are two prevalent approaches; Friedman is a differential method, each employing different approximations.^68^ This research employed model-free integral approaches to derive the values of *E*_a_ in conjunction with the original Vyazovkin method^62^

#### 2.4.1 Kissinger model

Kissinger introduced the initial model in 1956 for calculating activation energy through the non-isothermal thermogravimetric method.^69^ This approach is calculated for a specified conversion, under the assumption that the activation energy (*E*_a_) remains constant.^39^10
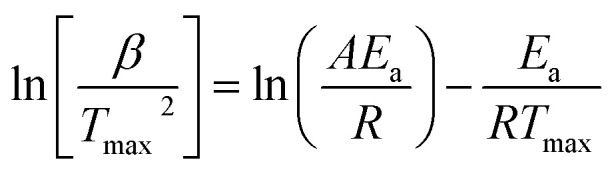
where, *T*_max_ represents DTG curve's peak temperature, while the heating rate is indicated by *β*. The activation energy (*E*_a_) was ascertained through plotting 
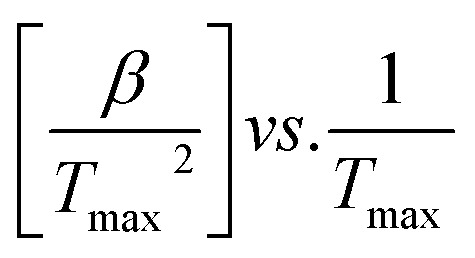
 for different heating rates (*β*).

#### 2.4.2 Ozawa–Flynn–Wall (FWO) model

The FWO model's equation is as follows:11
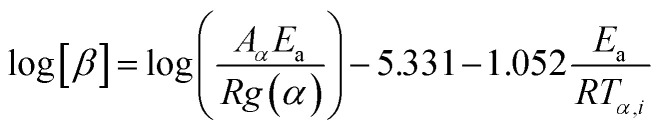
*g*(*α*) remains unchanged despite the conversion value in this context. The “*i*” and “*α*” subscripts represent a fractional conversion value and specific heating rate, respectively, in context of the equation. The slope of the log *β vs.* 1/*T* plots, which was −0.4567*E*_a_/*R*, was utilized in order to calculate *E*_a_ values for conversion values ranging from 0.1 to 0.9.^70^

#### 2.4.3 Kissinger–Akahira–Sunose (KAS) model

When studying biomass pyrolysis kinetics, the KAS model is often used as the most popular option. The pyrolysis kinetics are established by the KAS model using integral approaches. The Kissinger–Akahira–Sunose model can be expressed using the following equation.^71^12
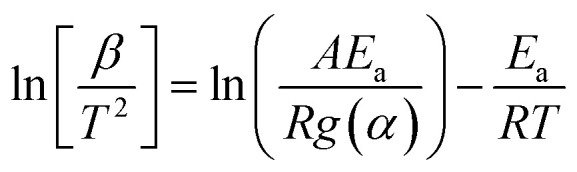


The graph between 
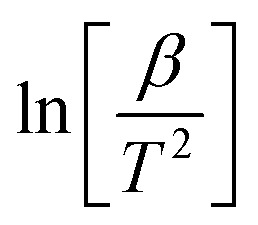
 and 
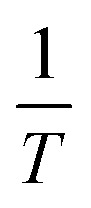
 at specific rates of conversion, with different heating rates, produces a linear relationship within the range of 0 to 1.^72^ Slope of the straight line obtained at varied conversion rates defines the process activation energy, and mean activation energies virtually fully characterize the reaction.^58^

#### 2.4.4 Friedman model



13

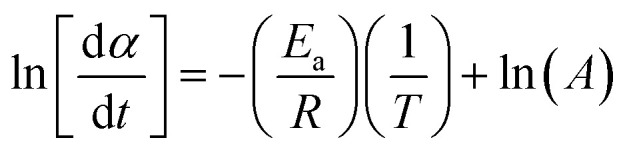




According to the isoconversional principle, eqn (13) may be treated as a linear equation at each specific conversion, thereby assuming constant *f*(*α*) and independent of reaction order. Subsequently, we would graph linear relationships of 
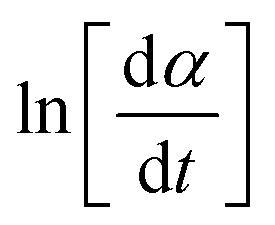
 against 
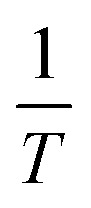
 at each individual conversion. The activation energy may be determined by calculating the slope of the line, while the pre-exponential factor can be determined by calculating the intercept of the line *via* the line.^73^ If we consider the fact that the intercept was supposed to stay unchanged, the pre-exponential component should not exhibit considerable variation with differing reaction orders.

#### 2.4.5 Vyazovkin model

The above techniques are complemented by more recent, advanced approaches. The proposed non-linear regression eqn (14) is used to obtain activation energy *via* the Vyazovkin approach. For calculating activation energy across a larger range of TGA data, this approach is acknowledged as being more accurate. In this manner, Vyazovkin's approach avoids the errors associated with the temperature integral's analytical approximation. *ϕ*(*E*_*y*_) represents finding a minimum value for eqn (14) where and, formula, the Vyazovkin method is applicable.

In the eqn (14), where (*E*_*α*_,*T*_*α*,*i*_) signifies the exponential integral and *n* denotes the number of heating rates, which may be computed using an approximation equation, A sophisticated isoconversional technique that uses the addition of *E*_α_ values to determine the activation energy is the Vyazovkin method calculated using Kinetic Calculation v. 1.0 program.^74^14
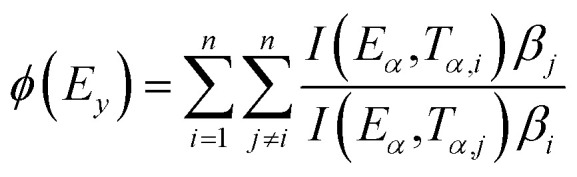


### 2.5 Thermodynamics analysis

The estimation of changes in Gibbs' free energy, entropy, and enthalpy are all components that are included in thermodynamic analysis calculations. Enthalpy is a measure that illustrates the difference in energy that exists between the feed complex and the activated complex.^75^ Equation computed as below (15)

15 Δ*H* = *E*_a_ − *RT*_m_

Gibbs free energy defines the spontaneity of a process.^76^ As the reaction progresses, the differential in Gibbs free energy reflects the residual energy within the system.^77^ It may be computed as follows using eqn (16)16
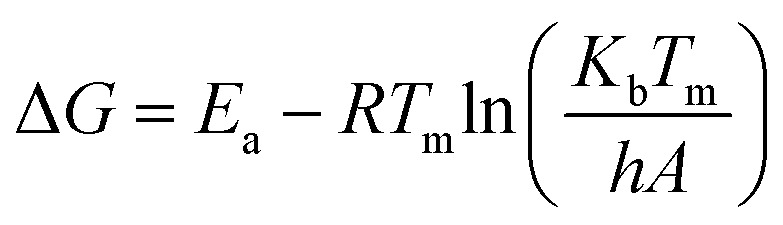


The degree of unpredictability or chaos that a system exhibit can be measured using its entropy.^78^ When the entropy value is low, it indicates that there is less reactivity in the system. The entropy value can be either positive or negative. It is possible to achieve the desired result by employing eqn (17). These measurements are helpful in determining whether or not the system is feasible and how much energy it contains.^79^17
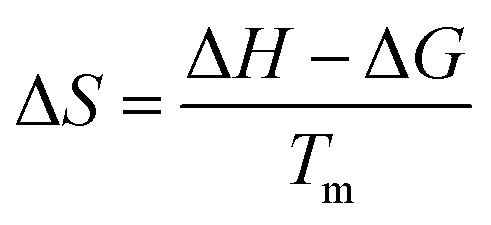
where, *h* = Planck constant (6.626 × 10^−34^ m^2^ kg s^−1^), and *K*_b_ = Boltzman constant (1.38 × 10^−23^ m^2^ kg s^−2^ k^−1^). For estimation of Δ*H*, Δ*S*, and Δ*G* at various conversions (*α*) for a heating rate of 10 °C min^−1^, the calculation was performed.^76^ Pre-exponential factor (*A*) given at eqn (16) calculated using the below eqn (18)18
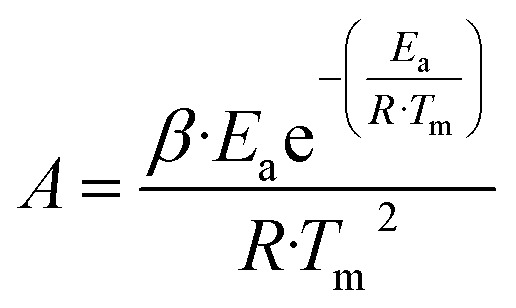


### 2.6 Equipment setup

**Fig. 1:**
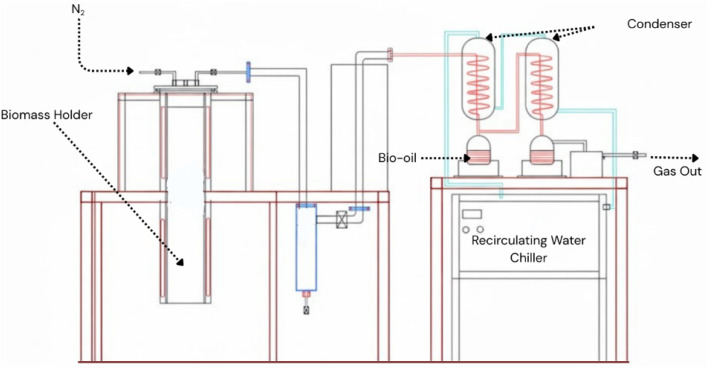
Schematic diagram of thermochemical conversion apparatus.

The pyrolysis experiments were conducted in a custom-designed vertical reactor system (Fig. 1). The biomass feedstock was loaded into a specialized sample holder. This assembly was positioned for thermal regulation during the volatilization process during slow pyrolysis (500 °C, 5 °C min^−1^). To maintain an inert reaction environment, nitrogen (N_2_) gas was introduced through a top-mounted inlet, serving as a carrier gas to sweep evolved volatiles from the main chamber. Downstream, the reactor was coupled to a multi-stage condenser and collection unit. Vapors excited the heated zones were routed through a dual-column condenser system connected to a recirculating water chiller, facilitating the condensation of bio-oil.

To predict and validate the product yields of the pyrolysis process, especially pyrogas, this study utilized a computational tool based on a Gradient Boosting Regressor (GBR) algorithm. The model was employed to forecast the quantitative distribution of bio-oil and syngas by analyzing the non-linear dependencies between specific feedstock characteristics and reaction parameters. Input variables, comprising biomass proximate and ultimate analyses alongside pyrolysis temperature, were entered into the interface to generate yield predictions, thereby leveraging the model's pre-trained capability to map H/C ratio, O/C ratio, pyrogas yield, oil yield, and biochar yield, which is utilized for techno-economic analysis.^80^ The SGSW experimental parameters were input into this pre-trained validated model to generate the theoretical baseline yields for comparison.^80^

### 2.7 Techno-economic analysis for economic feasibility

**Fig. 2:**
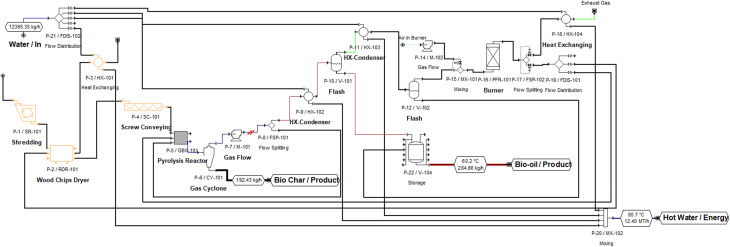
Process flow sheet of SGSW pyrolysis.

The predicted yield of biochar and biooil was taken from the GBR analysis. The techno-economic model for the co-production of biochar and bio-oil, leveraging experimental data, was developed using SuperPro Designer v13.1 software (Intelligen, Inc.) for a hypothetical industrial facility processing 0.33 MT h^−1^ of SGSW in India (Fig. 2). The model plant operated in continuous mode, with an annual operational duration of 330 days, excluding 35 days allocated for maintenance and cleaning. The project duration was established at 15 years. The construction and commissioning phases of the plant were anticipated to last 30 and 4 months, respectively. The procedure is modeled using a dynamic spreadsheet, estimating mass balance and energy consumption for pyrolysis and other unit processes, The corporate tax rate was maintained at 30% in accordance with Indian mandates, while the project's discount rate was established at 4%. The capital expenses were subsequently modified to align with the requisite capacities, utilising the power law. Assumptions are established regarding the costs of equipment categories, raw materials, wastewater treatment, heat transfer agents, and labour, as outlined in the GitHub repository. The economic assessment included the computation of capital expenditure (CAPEX) and annual operational expenses (OPEX).

To evaluate the project's financial feasibility and prospective returns, various critical metrics were analyzed, including gross margin (%), return on investment (ROI, %), payback period (in years), internal rate of return (IRR, %), and net present value (NPV, USD), as outlined in Sivaraman *et al.*^9^ Gross Margin. The sensitivity analyses were conducted to assess the impact of varying process scales (product throughput) on the process's profitability potential to achieve economic targets.^81^

### 2.8 Goal and scope of life cycle assessment for SGSW pyrolysis

**Fig. 3:**
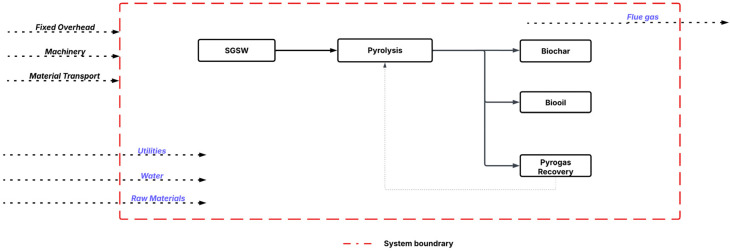
The boundary conditions for the life cycle assessment.

The primary objective of this Life Cycle Assessment (LCA) is to evaluate the environmental performance of industrial-scale biochar production utilizing *Sterculia guttata* shell waste (SGSW). This study identifies environmental hotspots and potential trade-offs within the thermochemical conversion process. The scope is defined as cradle-to-gate, encompassing all stages from the collection of SGSW (raw material acquisition) through the pyrolysis process to the final production of biochar. Downstream use (*e.g.*, soil application or filtration) and final disposal phases are excluded from the system boundary (Fig. 3).

### 2.9 LCA's allocation methodology

This study employs a mass-based allocation approach to distribute energy consumption and emissions among the output streams. This method operates on the premise that environmental burdens correlate directly with production volume. In the pyrolysis of SGSW, the process is multifunctional, yielding biochar. Recognizing that the industrial viability of SGSW conversion is driven by market demand, economic allocation is utilized as a secondary comparative measure. This method partitions environmental burdens according to the market value of the products.

### 2.10 LCA's inventory

Inventory analysis quantifies the resources and emissions for the SGSW-to-biochar system. The inventory utilizes primary data from Techno-Economic Analysis (TEA). Where specific data for*Sterculia guttata* collection is limited, the use of direct proxies as surrogate data in LCI is a feasible, robust, and practical approach to address data gaps.^82–85^ The SGSW pyrolysis assumed the required power would be drawn from the Indian average medium voltage electricity from the ecoinvent® 3.11 database.

### 2.11 LCA's methodology

The LCA model was developed in accordance with the International Reference Life Cycle Data System (ILCD) guidelines. Environmental impacts were characterized using the ReCiPe 2016 (Midpoint)(H) approach, which provides a comprehensive assessment across categories such as climate change, human toxicity, and resource depletion. Data processing and impact calculations were performed using OpenLCA 2.5.0, applying the ReCiPe 2016 normalization and weighting sets to identify significant environmental drivers.

## 3 Results and discussion

According to the proximate analysis, a large percentage of volatile matter was found to be present, which accounted for roughly 76.75 ± 0.35% of the SGSW biomass residues that were employed, the characteristics of the SGSW are listed in Table 1. The amount of fixed carbon was 10.14 ± 0.26%, whereas the amount of ash that was collected was 10.14 percent of the total biomass for the collection. The deconvolution of DTG data and compositional analysis of SGSW using the MixChar program revealed that lignin is the dominant constituent, accounting for 30.56 ± 1.66% of the biomass. Structurally, SGSW differentiates itself from commercial stone-fruit biomass as a dehiscent follicle rather than a sclereid-dense endocarp.^86^ Chemically, SGSW represents a high-density forestry residue with significant recalcitrance, positioning it as a premium carbon precursor superior to fibrous agricultural straws. This matches well with lignin content percentages for other shell waste biomass, including almond shells (20.4–28.4%), hazelnut shells (27.2–43.0%), and peanut shells (35.3%). This matches well with lignin content percentages for other shell waste biomass, including almond shells (20.4–28.4%), hazelnut shells (27.2–43.0%), and peanut shells (35.3%).^87^ Relatedly, Rohil Kumar *et al.*^88^ reported 36.48% of lignin for *S. foetida* shell biomass, which is similar to SGSW. This high lignin content suggests a strong potential for producing biochar with stable carbon structures and high calorific value, given lignin's resistance to thermal degradation and its aromatic, carbon-rich structure.^89^

**Table 1:** Main properties of SGSW biomass

Property	(wt%)
Volatile matter	76.75 ± 0.35
Moisture	2.84 ± 0.41
Fixed carbon	10.14 ± 0.26
Ash	10.19 ± 0.26
C	36.02
H	4.72
N	0.75
S	0.34
O	47.92
Cellulose	14.66 ± 6.08
Hemicellulose	11.47 ± 5.88
Lignin	30.56 ± 1.66

In contrast, cellulose (14.66 ± 6.08%) and hemicellulose (11.47 ± 5.88%) were present in lower proportions compared to typical lignocellulosic biomasses. The relatively lower cellulose and hemicellulose fractions may translate to a reduced yield of volatile products such as bio-oil and pyrogas during pyrolysis, as these components typically decompose at lower temperatures and contribute to volatile release.^90^

Pyrolysis was performed at the condition of 500 °C, 5 °C min^−1^, which yielded a distribution of products, biochar (34.20%), bio-oil (20.10%), and pyrogas, which was obtained from the difference. The produced biochar has enhanced carbon content (41.60%) (Table 2).

**Table 2:** SGSW pyrolysis yield and product characterizations

Product yield (wt%)			Composition (%)								
			Biochar				Bio-oil				
Biochar	Bio-oil	Pyrogas	C	H	N	S	Acids and alcohol	Aldehyde and ketone	Sugar and furans	Phenolics	Others
34.20	20.10	45.70	41.60	6.90	0.43	0.21	56.68	33.37	ND	2.76	7.19

Fan *et al.*^91^ observed that among the four raw shell biomass materials, such as the coconut shell, followed by the apricot shell, walnut shell, and peanut shell, the C content of the biochar samples post pyrolysis, especially >500 °C, has increased. This phenomenon is attributed to the dehydration, decarboxylation, and decarbonylation of the biomass that occur during the pyrolysis process, leading to the increase in C content as the volatile components are removed.^91,92^ The bio-oil composition obtained from GC-MS showed that acids/alcohols (56.68%) and aldehydes & ketones (33.37%) dominated. This product profile from SGSW pyrolysis is similar to that of other lignocellulosic biomasses, where these categories of compounds are dominant.^93–96^ To validate these experimental findings and assess the efficiency of the reactor setup, the experimental data were benchmarked against a predictive machine learning model trained on lignocellulosic feedstock composition.^80^ The model predicted distribution of 33.59 wt% biochar, 28.94 wt% bio-oil, and 35.99 wt% pyro-gas. The experimental biochar yield was strongly validated by the computational model, which predicted a theoretical yield. The comparison of the volatile fractions reveals that the experimental bio-oil recovery was lower than the model's theoretical prediction. This deviation highlights specific experimental constraints, specifically pointing to physical collection inefficiencies reported in the literature.^97–99^

**Fig. 4:**
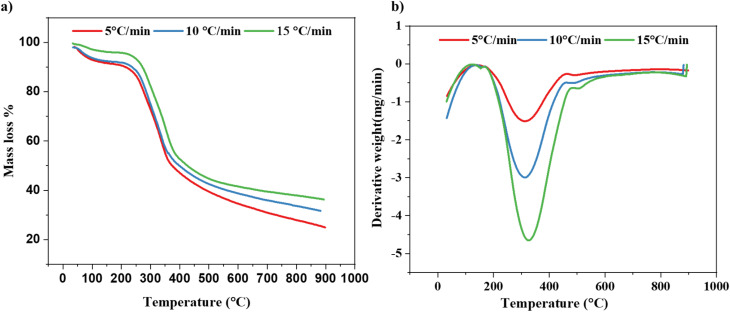
(a) TGA plot, (b) DTG plot of SGSW biomass performed at different rates of heating.

As a result of the heating rates, pyrolysis zones, which are passive and active, the ranges of temperature in the pyrolysis process, the peak temperature value of the DTG, and the amount of residual mass that is left over after the pyrolysis process is complete are all affected. Heating rates that are increased give adequate thermal energy, which in turn reduces the temperature disparity that exists between the interior and outer surfaces of biomass, which in turn promotes an increased conversion rate between the two types of surfaces. Furthermore, increasing heating rates results in a decreased response time for sample degradation at elevated temperatures, which causes temperature shifts towards higher values within the same temperature range, this is because the response time is lowered.^58^ According to the data presented in Fig. 4a, while heating rates at 5, 10, and 15 °C, corresponding peak temperatures of 309, 311, and 326 °C were observed, respectively. There is a correlation between the proportion of weight loss and the heating rate, with the proportion of weight loss decreasing as the heating rate increases. This association between the rates of heating and the percentage of weight reduction is depicted in Fig. 4b. At heating rates of 5, 10, and 15 °C min^−1^, respectively, within the temperature range of 200 to 700 °C, the percentages of weight loss are 93%, 85%, and 83%, respectively. As shown in Fig. 4a, the percentage of residual weight remains the same across different rates of heating. This suggests that the total weight loss is uniform and that the process is driven by a single mechanism that is independent of the heating rates.

**Fig. 5:**
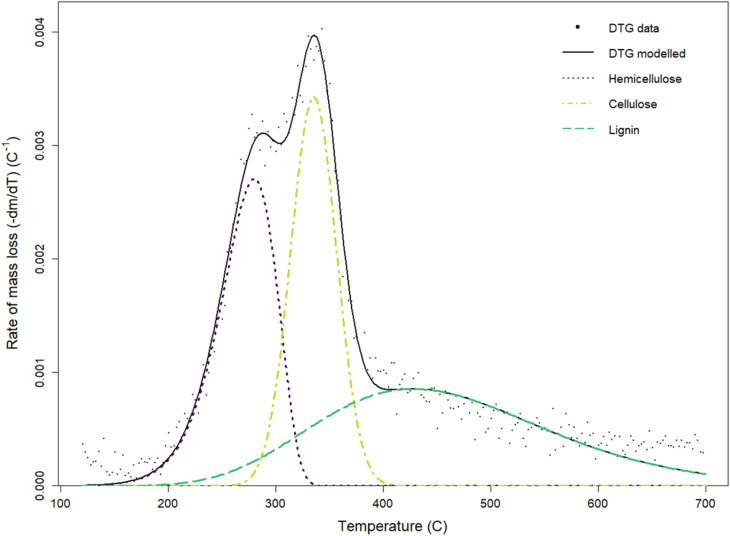
Deconvolution of DTG data (10 °C min^−1^) of using part Fraser–Suzuki mixture model fitted from 120 °C to 700 °C.

Rapid heating and brief residence times improve the release of volatile compounds, thereby augmenting bio-oil yield during the biomass pyrolysis.^66^ The pyrolysis of lignocellulosic biomass consists of three main stages: moisture removal (at 140 °C), hemicellulose degradation (at 200–350 °C), and cellulose breakdown (at 250–500 °C), followed by lignin decomposition (at 550 °C). Lignin constitutes the predominant component, with 30.56 ± 1.66% of the biomass. The elevated lignin concentration indicates a significant potential for generating biochar characterized by stable carbon structures and high calorific value, owing to lignin's resilience to heat degradation and its aromatic, carbon-dense composition. Conversely, cellulose (14.66 ± 6.08%) and hemicellulose (11.47 ± 5.88%) were found in lesser quantities relative to standard lignocellulosic biomasses. The diminished cellulose and hemicellulose fractions may result in a decreased output of volatile compounds, such as bio-oil and pyrogas, during pyrolysis, as these components generally disintegrate at lower temperatures and facilitate volatile release (Fig. 5).^100^

**Fig. 6:**
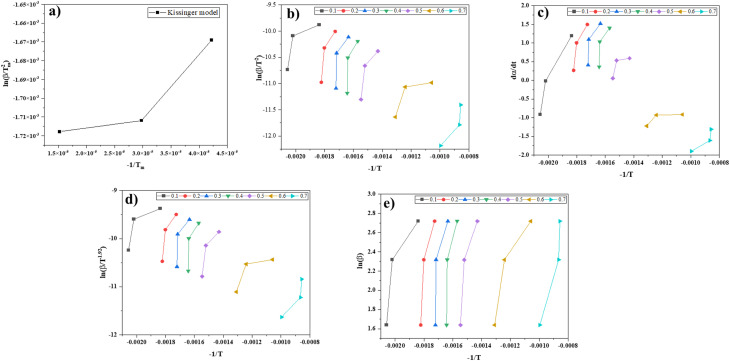
Estimation of activation energy using various isoconversional methods (a) Kissinger method (b) KAS method (c) Friedman method (d) Starink method (e) FWO method.

The biomass pyrolysis of SGSW residue's kinetic parameters were calculated using approaches of isoconversional model-free, including KAS, Kissinger, Friedman, FWO, and Vyazovkin. The International Confederation for Thermal Analysis and Calorimetry (ICTAC) has proposed isoconversional techniques for calculating kinetic parameters using thermogravimetry data related to the thermal degradation of carbonaceous materials.^101^Tables 3 and 4 present the variation in energy activation relative to conversion (*α*) for all model-free isoconversional methods examined. The slopes of linear plots serve to generate the activation energy estimations obtained from the assessed isoconversional procedures^102^ (Fig. 6).

**Table 3:** Kinetic parameter calculations of SGSW – KAS, FWO and Starink models

*α*	KAS			FWO			Starink		
	*E*_a_ (kJ mol^−1^)	*A* (min^−1^)	*R* ^2^	*E*_a_ (kJ mol^−1^)	*A* (min^−1^)	*R* ^2^	*E*_a_ (kJ mol^−1^)	*A* (min^−1^)	*R* ^2^
0.1	25.03	1.57 × 10^1^	0.635	31.95	8.33 × 10^1^	0.759	25.18	1.62 × 10^1^	0.641
0.2	70.80	5.57 × 10^5^	0.759	76.22	1.84 × 10^6^	0.802	70.61	5.34 × 10^5^	0.761
0.3	65.68	1.8 × 10^5^	0.589	71.87	7.05 × 10^5^	0.655	65.56	1.75 × 10^5^	0.592
0.4	79.80	4.02 × 10^6^	0.602	85.70	1.46 × 10^7^	0.659	79.57	3.83 × 10^6^	0.604
0.5	55.06	1.69 × 10^4^	0.746	62.98	9.89 × 10^4^	0.810	55.06	1.69 × 10^4^	0.749
0.6	17.94	2.6 × 10^0^	0.615	30.52	5.93 × 10^1^	0.842	18.36	2.9 × 10^0^	0.630
0.7	37.21	2.87 × 10^2^	0.807	52.43	9.33 × 10^3^	0.901	37.63	3.17 × 10^2^	0.812
Avg.	50.21			58.80			50.28		

**Table 4:** Kinetic parameter calculations of SGSW – Friedman and Vyazovkin models

*α*	Friedman			Vyazovkin	
	*E*_a_ (kJ mol^−1^)	*A* (min^−1^)	*R* ^2^	*E*_a_ (kJ mol^−1^)	*A* (min^−1^)
0.1	71.85	7.03 × 10^5^	0.925	53.57	1.21 × 10^4^
0.2	92.28	6.1 × 10^7^	0.829	70.86	5.64 × 10^5^
0.3	78.44	2.98 × 10^6^	0.667	71.75	6.87 × 10^5^
0.4	85.74	1.47 × 10^7^	0.641	77.48	2.42 × 10^6^
0.5	29.84	5.03 × 10^1^	0.558	87.02	1.94 × 10^7^
0.6	8.13	1.56 × 10^−1^	0.536	82.77	7.7 × 10^6^
0.7	27.77	3.06 × 10^1^	0.787	59.64	4.7 × 10^4^
Avg.	56.29			71.86	

The heterogeneous content of biomass fibers, on the other hand, causes thermal breakdown to take place throughout a wide temperature spectrum, which in turn causes the activation energy to be vary.^78^*E*_a_ is affected by factors such as the composition of the fiber, the types of fuel, mathematical calculations, and the operational parameters of the pyrolysis process.

The activation energies that were computed using the slope that was discovered from the equations at various conversions (*α*) are presented in Table 4. These activation energies were reported in for the, Starink, FWO and KAS approaches. Table 4 demonstrates that the correlation coefficient (*R*^2^) values for the models that were utilized in the process of calculating activation energy are often close to 1. This indicates that these models are reliable in calculating the activation energy of the SGSW biomass.^103^ According to Kirti *et al.*,^76^ the pyrolysis of agricultural residue is characterized by its complex nature, heterogeneity, and multi-step reaction mechanism. This insight is revealed by the rise and reduction in *E*_a_ that occurs throughout conversion. According to the results of pyrolysis, the reduction of the *α*, the *E*_a_ increased between 0.1 and 0.4. This is because cellulose has become the most significant constituent after the reduction.^104^ However, the *E*_a_ decreased at conversion rates between 0.5 and 0.7, which was the final phase. A lower activation energy resulted from lignin having achieved thermal equilibrium for pyrolysis.^104^ The KAS, FWO and Starink procedures were used to calculate the average activation energy throughout the entire conversion range.^105^ The results showed that the KAS approach yielded 50.21 kJ mol^−1^, meanwhile, 58.8 kJ mol^−1^ was shown by FWO method, whereas the starink model yielded 50.28 kJ mol^−1^. The results obtained from the various models do not differ significantly from one another.^106^ According to Mishra and Mohanty^107^ the energy content of waste biomass *Azadirachta indicum* was 176.66 kJ mol^−1^ for the KAS method and 193.67 kJ mol^−1^ for the FWO model simultaneously.^107^ Previous research conducted by Sriram *et al.*^61^ determined that the activation energy of *Musa balbisiana* was 138.2 kJ mol^−1^ when using the Friedman model. This value is consistent with the above findings. The model-free isoconversional methods, Friedman and Vyazovkin, yielded lower average activation energies of 56.29 kJ mol^−1^ and 71.86 kJ mol^−1^, respectively. These lower values are consistent with the differential nature of these models, which consider instantaneous reaction rates and are more sensitive to early-stage devolatilization processes.^106,108^ The Vyazovkin model, a non-linear isoconversional method, was employed to analyze the pyrolysis kinetics of SGSW, demonstrating superior accuracy in capturing the multi-stage decomposition of this lignin-rich biomass.

**Fig. 7:**
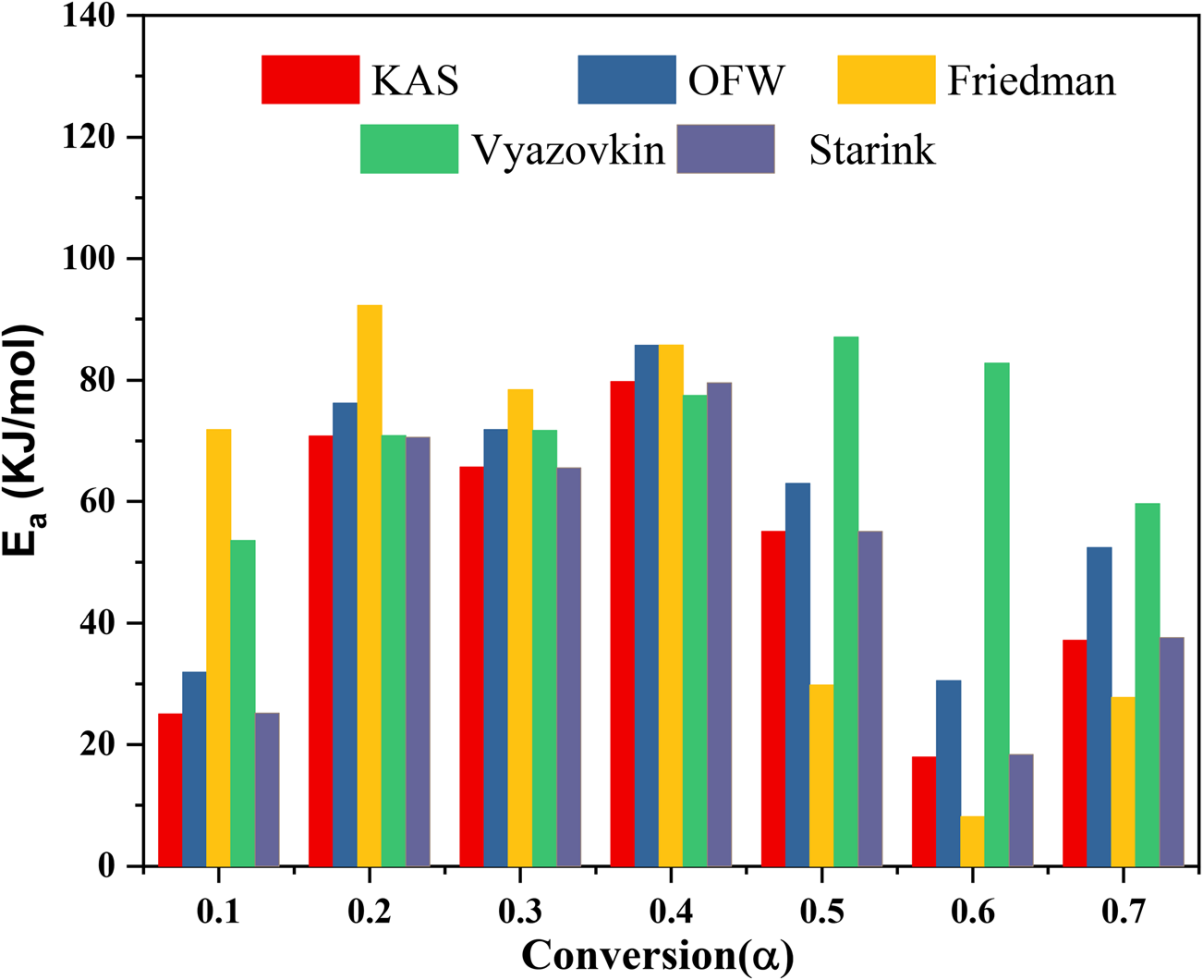
Variation of activation energy with respective conversion by different models.

For SGSW, Fig. 7 shows that the Vyazovkin method yielded an average *E*_a_ of 71.86 kJ mol^−1^, significantly higher than values from Kissinger–Akahira–Sunose (50.21 kJ mol^−1^) and Flynn–Wall–Ozawa (58.8 kJ mol^−1^).^65^ The *E*_a_ varied with conversion, rising from 53.57 kJ mol^−1^ at *α* = 0.1 to 87.02 kJ mol^−1^ at *α* = 0.5, reflecting the dominance of lignin decomposition (30.56% of biomass) at higher temperatures.^109^ This contrasts with cellulose/hemicellulose degradation, which occurs at lower, more consistent *E*_a_. The pre-exponential factor (*A*) spanned 10^4^–10^7^ min^−1^, aligning with lignin's complex, multi-pathway breakdown.^110^

The average activation energy (*E*_a_) determined *via* the Friedman method was 56.29 kJ mol^−1^, which is lower than that derived from the Vyazovkin method (71.86 kJ mol^−1^) but higher than values obtained from the KAS (50.21 kJ mol^−1^) and Starink (50.28 kJ mol^−1^) methods.^111,112^ Notably, *E*_a_ varied significantly with the extent of conversion (*α*), ranging from 92.28 kJ mol^−1^ at *α* = 0.2 to as low as 8.13 kJ mol^−1^ at *α* = 0.6 (as shown in Table 4). This variation reflects the multi-stage decomposition behavior of SGSW's lignocellulosic structure composed of cellulose and lignin with lignin playing a dominant role at higher conversion stages.^103^ The *E*_a_ obtained from the Friedman method was approximately 20% higher than those from the KAS and Starink methods, but about 22% lower than that from the Vyazovkin method.^113^ This discrepancy stems from the methodological differences: Friedman, as a differential method, captures instantaneous reaction rates and is particularly sensitive to early-stage devolatilization, whereas integral methods like Vyazovkin average kinetic effects over broader temperature ranges.^114^ One of the major strengths of the Friedman method is its model-free nature, making it well-suited for heterogeneous biomass like SGSW. Additionally, it effectively captures the rapid devolatilization kinetics of hemicellulose and cellulose at lower conversions (*α* < 0.5), offering more accurate insights than integral approaches.^115^ Thermodynamic analysis using Vyazovkin-derived *E*_a_ revealed an average enthalpy change (Δ*H*) of 54.8 kJ mol^−1^, confirming the endothermic nature of pyrolysis.^113^ Gibbs free energy (Δ*G*) ranged from 151.73 to 156.81 kJ mol^−1^, indicating non-spontaneous reactions requiring external energy input.^116^ Negative entropy values (−0.12 to −0.18 kJ mol^−1^ K) suggested increased order in the activated complex.

Vyazovkin model precision in capturing SGSW's multi-stage kinetics highlights its advantage over differential methods like Friedman, which are more sensitive to experimental noise.^18^ Its model-free nature and ability to handle parallel reactions make it particularly suited for lignin-rich biomass, providing critical insights for optimizing bioenergy production from underutilized SGSW.

The activation energies obtained from FWO, KAS, Starink, Friedman, and Vyazovkin methodologies were used to calculate the thermodynamic parameters such as Δ*H*, Δ*S*, and Δ*G*, as outlined in Tables 4 and 5, using eqn (15)–(17).^103,117^ The complex composition of the biomass sample and the diverse processes that occur during decomposition can be attributed to the variation in pre-exponential factor during conversion.^118^Eqn (16) yields an *A* (min^−1^) value of ranging 10^6^–10^7^ min^−1^, as illustrated in Tables 4 and 5. The KAS FWO, Starink, Friedman, and Vyazovkin approaches pertain to the frequency of collisions between reactant molecules and the nature of the resulting complex.^119^ This suggests that the pyrolysis of SGSW agro-residue is a multifaceted process.^120^ Particle collision in biomass is significant when *A* is between 10^10^ and 10^12^ min^−1^, while only surface reactions occur when A is less than 10^9^ min^−1^. A number exceeding 10^12^ min^−1^ signifies that vigorous molecular collisions are necessary, suggesting that biomass pyrolysis demands substantial activation energy. In this application, enthalpy denotes the amount of energy or heat required for the transformation of biomass into various products.^39^ The isoconversional methods produce average enthalpy values between 22.9 kJ mol^−1^ and 54.8 kJ mol^−1^.

**Table 5:** Thermodynamic parameters calculation of SGSW pyrolysis – KAS, OFW and Starink models

*α*	KAS			FWO			Starink		
	Δ*H* (kJ mol^−1^)	Δ*G* (kJ mol^−1^)	Δ*S* (kJ mol^−1^ K^−1^)	Δ*H* (kJ mol^−1^)	Δ*G* (kJ mol^−1^)	Δ*S* (kJ mol^−1^ K^−1^)	Δ*H* (kJ mol^−1^)	Δ*G* (kJ mol^−1^)	Δ*S* (kJ mol^−1^ K^−1^)
0.1	20.18	157.77	−0.24	27.09	156.59	−0.22	20.32	157.74	−0.24
0.2	65.94	152.73	−0.15	71.37	152.37	−0.14	65.75	152.74	−0.15
0.3	60.83	153.10	−0.16	67.01	152.66	−0.15	60.70	153.10	−0.16
0.4	74.94	152.15	−0.13	80.84	151.81	−0.12	74.72	152.17	−0.13
0.5	50.20	153.95	−0.18	58.12	153.30	−0.16	50.21	153.95	−0.18
0.6	13.08	159.39	−0.25	25.67	156.81	−0.22	13.50	159.28	−0.25
0.7	32.36	155.85	−0.21	47.57	154.19	−0.18	32.77	155.80	−0.21
Avg.	45.36	154.99	−0.19	53.95	153.96	−0.17	45.42	154.97	−0.19

The pyrolysis enthalpy values for SGSW biomass were determined to be positive and showed a decrease from 0.1 to 0.4 conversion. The pyrolysis of SGSW biomass is an endothermic process; however, the endothermicity of the process declines as the conversion rate increases.^121^ A study by White *et al.*^28^ also observes this phenomenon. The average Gibbs free energy was calculated by the KAS FWO, Starink, Friedman, and Vyazovkin methods to be between 152.1 kJ mol^−1^ and 156.8 kJ mol^−1^. These methods suggest that the Δ*G* obtained from these two methodologies is comparable and consistent. The system's nearly constant Δ*G* values point to an overestimation of the heat transfer and a positive reaction mechanism.^72^ Kirti *et al.*^76^ demonstrated similar findings regarding the pyrolysis of *Cajanus cajan* stalk. One measure of systemic disorder is the entropy, a state function, a low “*S*” value in a system means that the material has recently experienced a chemical or physical change that has produced its current condition.^39^ Nearing thermodynamic equilibrium, a novel states the chemical's restricted reactivity in this scenario extends the time necessary to produce an active molecule. A larger ‘*S*’ value, conversely, indicates that the substance is thermodynamically out of equilibrium.^122^ Consequently, the response system will exhibit rapid reaction in this scenario due to its elevated responsiveness, the production of the activated complex leads to shortened response times.^123^

According to the thermodynamic analysis, the pyrolysis of SGSW agro residue was endothermic, necessitated the creation of complexes, required reduced energy, and reached thermodynamic equilibrium.^76^ According to the results, SGSW biomass could be a good source of bioenergy products (Tables 5 and 6).

**Table 6:** Thermodynamic parameters calculation of SGSW pyrolysis – Friedman and Vyazovkin models

*α*	Friedman			Vyazovkin		
	Δ*H* (kJ mol^−1^)	Δ*G* (kJ mol^−1^)	Δ*S* (kJ mol^−1^ K^−1^)	Δ*H* (kJ mol^−1^)	Δ*G* (kJ mol^−1^)	Δ*S* (kJ mol^−1^ K^−1^)
0.1	67.00	152.66	−0.15	48.71	154.08	−0.18
0.2	87.42	151.45	−0.11	66.00	152.73	−0.15
0.3	73.58	152.24	−0.13	66.89	152.67	−0.15
0.4	80.88	151.80	−0.12	72.63	152.29	−0.14
0.5	24.98	156.92	−0.23	82.16	151.73	−0.12
0.6	3.27	163.23	−0.27	77.92	151.97	−0.13
0.7	22.91	157.27	−0.23	54.79	153.56	−0.17
Avg.	51.43	155.08	−0.18	67.01	152.72	−0.15

The isoconversional model-free methodologies employed in this investigation have the capacity to produce results of satisfactory precision. Komandur *et al.*^124^ contend that the kinetic parameters derived from model-free isoconversional methods are superior for generating initial estimates for fitting kinetic models, acknowledging that pyrolysis is a multifaceted process comprising several reactions rather than a singular reaction step. Different interpretations of isoconversional data can yield diverse results. The assumptions that were made throughout the process of deriving integral techniques are a significant source of inaccuracy and a major topic of contention in the field.^19^

Isoconversional techniques are model-free, which means that they do not make any predictions regarding the reaction mechanism or the preexponential component itself. This is an additional point of contention. Kinetic studies are essential for reactor design, process optimization, and predicting product yields and composition. They provide key data for scaling up processes from laboratories to industrial levels and for understanding the thermochemical behavior of various feedstocks.^125–128^ Technoeconomic Analysis (TEA) is crucial for evaluating the commercial potential of pyrolysis technologies, identifying technical and economic barriers, and guiding process integration with existing systems.^127,129,130^

The simulated pyrolysis plant is designed to examine the techno-economic feasibility of scaling up lab-scale pyrolysis data to an industrial scale for the conversion of SCSW into biochar. The pyrolysis modelling detailed in Fig. 2 utilizes a comprehensive techno-economic analysis by calculating capital investment, manufacturing costs, and other economic indicators (Table 7). The process operates to produce biochar as the main revenue stream, with an annual production capacity of approximately 875 metric tonnes. In addition to the primary product, the facility recovers valuable by-products, specifically producing 131 008 kg of bio-oil and 34 445 MT of hot water annually. As indicated by the simulation data, the total Capital Expenditure (CAPEX) for the proposed facility is projected to be USD 2.03 million, with a Direct Fixed Capital (DFC) investment of USD 1.89 million and a startup cost of USD 95 000.

**Table 7:** Techno-economic evaluation summary of biochar production from pyrolysis of SGSW

Description	Value	Units
Direct fixed capital	1 893 000	$
Working capital	37 000	$
Startup cost	95 000	$
CAPEX	2 025 000	$

**Production rates**		
Bio char (main revenue)	875 000	kg per year
Hot water (revenue)	34 445	MT per year
Bio-oil (revenue)	1 131 008	kg per year

**Revenue rates from products**		
Bio char (main revenue)	1.19	$ per kg
Hot water (revenue)	1.00	$ per MT
Bio-oil (revenue)	0.54	$ per kg

**Total revenues**		
Bio char (main revenue)	1 041 667	$ per year
Hot water (revenue)	34 445	$ per year
Bio-oil (revenue)	605 897	$ per year
Total revenues	1 682 009	$ per year

**Annual operating cost**		
Net annual operating cost (OPEX)	923 000	$ per year

**Unit production cost/revenue**		
Unit production cost	1.05	$ per kg MP
Net unit production cost	1.05	$ per kg MP
Unit production revenue	1.92	$ per kg MP

**Profitability indicators**		
Net profit	787 000	$ per year
Gross margin	45.13	%
Return on investment	38.87	%
Payback time	2.57	years
IRR (after taxes)	49.81	%
NPV (at 3.0% interest)	6 678 000	$

Brown,^131^ finds that the choice of pessimistic or optimistic capital cost estimation methodology can change the NPV by up to $300 million for the analyzed biofuel pathways. The Total Capital Investment (TCI) for 1 MT h^−1^ corn stover biochar plants, the TCI is estimated to be approximately $320 000.^132^ Swanson *et al.*^133^ calculated the TCI for a biomass-to-liquid fuel production plant in the range of $500–650 million. This indicates the scale of the plant widely varies for biomass-based technologies. Mariselvam *et al.*^134^ identifies total capital costs as largely attributed to plant direct costs, with equipment costs and installation being significant contributors to the expenses for biochar production.

The economic evaluation highlights a total annual revenue of USD 1.68 million, driven primarily by biochar sales at USD 1.19 per kg, supplemented by bio-oil (USD 0.54 per kg) and hot water (USD 1.00 per MT). Consequently, the unit production cost is estimated at USD 1.05 per kg of the main product, compared to a unit revenue of USD 1.92 per kg, resulting in a gross margin of 45.13%. Profitability indicators suggest a highly viable investment, with a projected Net Profit of USD 787,000 per year. The project demonstrates a Return on Investment (ROI) of 38.87% and an estimated payback period of 2.57 years.

The facility's gross margin of 45.13% compares favorably to the typical industry average.^135^ Furthermore, the Net Present Value (NPV) is estimated at USD 6.68 million (at 3.0% interest), confirming the significant economic efficiency of valorizing SCSW through pyrolysis. Mariselvam *et al.*^134^ performed NPV analysis for sugarcane bagasse biochar production, suggesting positive investment feasibility, with a net payback time of 7.33 years and a rate of return on investment reaching a maximum of 16.83% after 11 years.

**Fig. 8:**
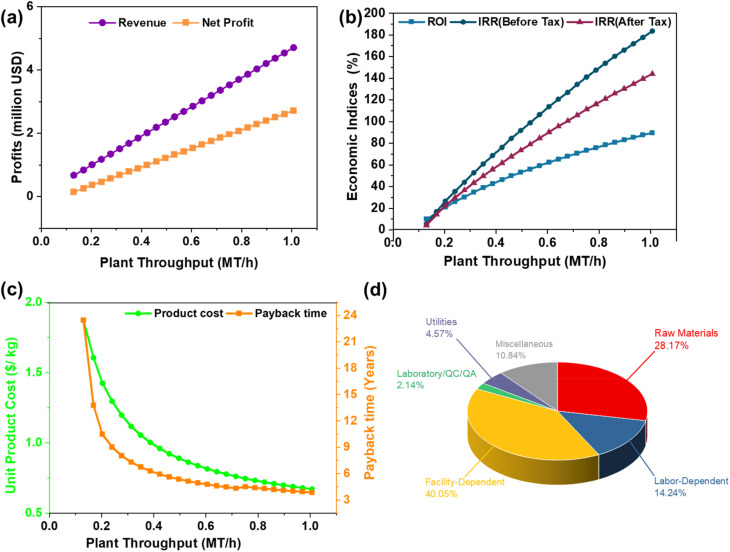
Effect of plant capacity on (a) annual revenues and net profits, (b) economic indices (ROI and IRR), and (c) effect of batch throughput on payback time (years) and unit production cost. (d) Annual operating cost breakdown.

The Net Annual Operating Cost (OPEX) was calculated at USD 923 000. Fig. 8d illustrates the breakdown of annual operating costs, highlighting the capital-intensive nature of biochar production processes, where facility-dependent costs account for 40.08% of total annual expenses. Ebrahimian and Mohammadi^136^ studied production of 2,3-butanediol (BDO) from wood residues, found that facility-dependent cost contributed 33% of the total operating cost in the studied biorefinery scenarios. Similarly, Baral and Shah^137^ studied fast pyrolysis of stillage from a cellulosic biorefinery, found that facility-dependent costs are 35.1%, which were a major contributor to the operating costs. This is due to expenses tied to maintaining and running the physical plant and equipment, including depreciation, taxes, insurance, and overhead.^136–138^ This is followed by raw materials (28.1%) and labour (14.24%) costs. In TEA of waste valorization, it is common to assume a zero-opportunity cost for raw materials, especially when the feedstocks are agricultural or industrial wastes.^139,140^ Assuming zero raw material costs can lead to overly optimistic estimates of commercial viability and profitability, as it may not account for real-world expenses such as collection, transportation, and preparation of feedstocks.^141,142^ Given that SGSW, which is primarily collected from *Sterculia* sp., holds socio-cultural importance among tribal communities, the raw material cost of 0.09 USD/Kg is assumed in this study.^16,86^ The labour-dependent cost is relatively low; this is due to India's labour cost in manufacturing being significantly lower than that in developed economies.^143–145^

Fig. 8a demonstrates that annual revenues and net profits increase linearly with plant throughput. Unlike batch processes, where scheduling bottlenecks may plateau revenue, the continuous mode allows for linear scaling up to 1.0 MT h^−1^. The break-even point is observed at a low throughput of approximately 0.15 MT h^−1^, where the net profit shifts from negative to positive. The sensitivity of investment metrics to throughput is further detailed in Fig. 8b. The Return on Investment (ROI) and Internal Rate of Return (IRR) display a non-linear, upward trajectory. The ROI surpasses the strategically sound 20% threshold at a throughput of roughly 0.2 MT h^−1^ and continues to rise, reaching nearly 90% at 1.0 MT h^−1^. This suggests that while the initial capital investment for continuous pyrolysis equipment is substantial, maximizing the continuous feed rate is essential for ensuring economic viability and shortening the payback period. To increase production capacity further, additional capacity would be required, which would increase CAPEX step-wise, but within the simulated range, higher throughput correlates strongly with improved economic efficiency. The ROI threshold of 20% is strategically sound for bioprocess ventures.^146^ To increase production capacity, it is necessary to acquire more equipment to eliminate scheduling bottlenecks. However, as mentioned before, the investment would increase considerably.

As illustrated in Fig. 8c, the unit product cost exhibits a sharp exponential decay as plant throughput increases from 0.1 to 0.4 MT h^−1^, eventually stabilizing as the throughput approaches 0.8 to 1.0 MT h^−1^. This stabilization indicates the achievement of economies of scale, similar to findings in comparable bioprocessing operations, where scaling up capacity significantly mitigates unit costs.

**Fig. 9:**
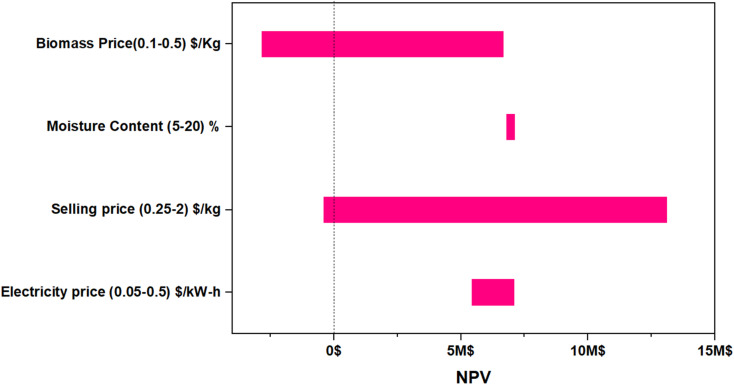
Impact of critical parameters of SGSW pyrolysis on the NPV at 3% interest.

The sensitivity analysis illustrated in Fig. 9 demonstrates biochar selling price has the most substantial impact on the economic feasibility of SCSW pyrolysis. Sensitivity analyses across multiple studies confirm that NPV is highly sensitive to feedstock cost, along with product selling price and capital investment. While feedstock cost and plant scale can influence MSP and NPV, the selling price of the final product remains the most sensitive and decisive factor in determining project profitability. Feedstock cost and plant scale can influence MSP and NPV, the selling price of the final product remains the most sensitive and decisive factor in determining project profitability. Alonso-Gómez *et al.*^147^ found that cassava residue-based biochar plant, a minimum selling price of 1732 USD per ton was required to reach equilibrium at a certain processing scale and increasing the selling price improved economic indicators. Qi *et al.*^148^ in a molten salt heating tire pyrolysis process, a 20% increase in pyrolysis oil price reduced the payback time from 5.87 to 4.51 years. A narrow spread in moisture prices indicated a relatively lower percentage of operating costs associated with medium variables in the production process. Additionally, the notable influence of utility expenses, including electricity. Similarly, Bajić *et al.*^149^ highlighted that energy demands for fermentation and downstream processing played a decisive role in determining overall economic viability. Sensitivity analysis indicates that production scalability and biomass allocation between different product streams have a significant impact on profitability metrics.^9^

**Fig. 10:**
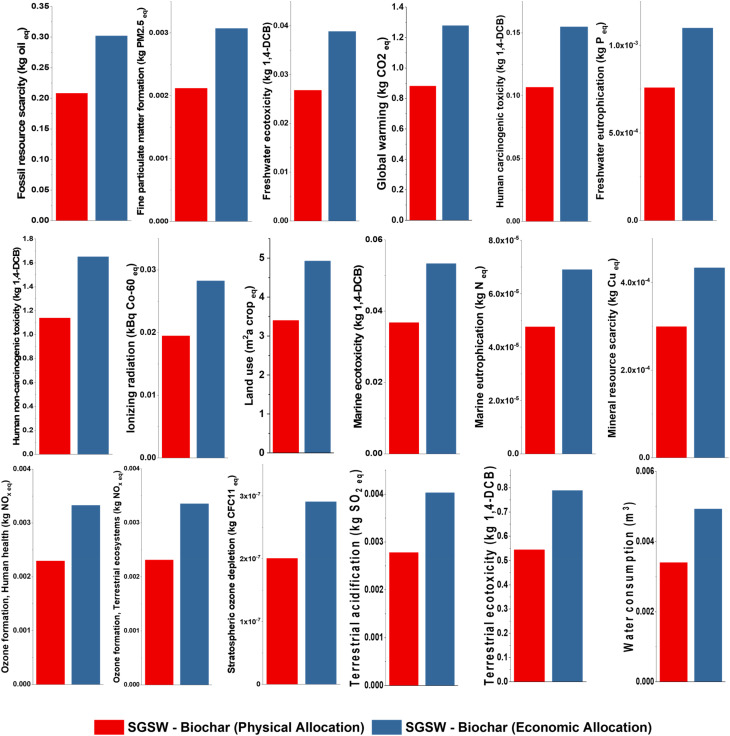
Life cycle impact assessment of the production of 1 kg of SGSW biochar.

To move toward a circular bioeconomy, we need to turn agricultural waste into useful goods like biochar, bio-oil, and pyrogas. Among the new feedstocks, SGSW is a great candidate for thermochemical conversion since it has a high carbon density and a lignocellulosic structure. Nonetheless, the environmental sustainability of these systems depends on the scientific rigor of the Life Cycle Assessment (LCA). Fig. 10 offers a comprehensive examination of the environmental profile of SGSW pyrolysis, focusing on the responsiveness of 18 ReCiPe Midpoint effect categories to allocation strategies.

This study delineates the environmental trade-offs associated with the recovery of bioresources within the Indian industrial context, employing primary data from TEA and secondary data from the ecoinvent 3.11 database. The Indian energy sector remains heavily reliant on fossil fuels, with coal-based thermal power plants accounting for over 51.7% of the total installed capacity.^150^ This dependency exerts a significant influence on the LCA results, as the combustion of hard coal is a primary driver of global warming, acidification, and human toxicity.^151^ The ecoinvent 3.11 database incorporates the latest statistics on methane emissions from coal mining and the specific fuel efficiencies of Indian power plants, providing a high-fidelity representation of the upstream impacts associated with utility consumption in the pyrolysis facility.^152^ The Global Warming Potential (GWP) is recorded at 1.27 kg CO_2_ eq under economic allocation, compared to 0.88 kg CO_2_ eq for physical allocation. These values are competitive when compared to other biomass sources, such as date palm waste (1.53 kg CO_2_ eq per kg).^153^ The production-phase GWP is primarily driven by the energy required for shredding and machinery operation. In the Indian context, where the electricity grid remains over 50% reliant on coal-based thermal power plants, the carbon footprint of utility consumption is high.^154^ Hachicha *et al.*^155^ found that net GWP of biochar produced from forest residues can be as low as 0.1–1.6 kg CO_2_-eq per kg of biochar. Puettmann *et al.*^156^ estimated that 0.53 kg CO_2_ eq GWP for portable biochar systems using forest residues when not accounting for carbon credits or avoided emissions from alternative residue disposal. Eduardo *et al.*^157^ states that forestry residue biochar can have a GWP of 1.00 kg CO_2_-eq per kg of biochar before sequestration benefits.

Pyrolysis also results in lower emissions of harmful gases (NO_*x*_, SO_2_) and ecotoxicity compared to combustion and landfilling.^158^ Human Carcinogenic Toxicity of 1.54 kg 1,4-DCB for SGSW biochar is primarily influenced by the emission of heavy metals such as arsenic and chromium during the life cycle of coal power. Similarly, Chen *et al.*^159^ found that bamboo biochar production in a co-production system with hydrogen 0.22 kg 1,4-DCB eq. Fine Particulate Matter Formation of 0.003 kg PM 2.5 eq is particularly sensitive to the Indian context, where thermal power plants are significant sources of secondary particulates from SGSW biochar production.^160^ Anand *et al.*^161^ reported for 69.0 MT coal equivalence of biochar annually could reduce PM emissions by 0.19 MT (including PM_2_._5_ and PM_10_), along with reductions in SO_2_, NO_*x*_, CO, VOCs, and CO_2_. The majority of PM_2_._5_ emissions from coal-based electricity arise from both combustion and upstream supply chain activities, highlighting the importance of a full life cycle perspective.^152^ Fossil resource scarcity follows a similar trend, by showing 0.302 kg oil eq. Land use impacts are quantified at 4.9 m^2^ crop eq for economic allocation and 3.3 m^2^ crop eq for physical allocation. While SGSW biochar production is a sustainable waste valorization strategy, its environmental profile is heavily dictated by the socio-economic value of the products and the carbon intensity of the national energy grid. The use of internal pyrogas recovery remains the most effective mitigation strategy for improving the system's net sustainability.

Further research should concentrate on improving the circumstances of the pyrolysis process in order to improve the amount and quality of the products that are being targeted (bio-oil, biochar, or syngas) in accordance with the kinetic and thermodynamic characteristics that have been outlined in this work. It is also possible that research into the catalytic pyrolysis of SGSW could further reduce the amount of energy that is required for activation and increase product selectivity. In order to evaluate the commercial potential and environmental sustainability of SGSW as a feedstock for bioenergy, it is essential to conduct life cycle assessments pertaining to the material viability and environmental sustainability of SGSW as a bioenergy feedstock.^162^

The transition toward a circular bioeconomy necessitates the valorization of agricultural residues into value-added products such as biochar, bio-oil, and pyrogas. Among the emerging feedstocks, SGSW presents a significant opportunity for thermochemical conversion due to its high carbon density and lignocellulosic structure. However, the environmental sustainability of these systems is contingent upon the methodological rigor of the LCA.

## 4 Conclusion

This study presents the first comprehensive valorization framework for *Sterculia guttata* shell waste (SGSW), uniquely integrating isoconversional kinetic modeling with Techno-Economic Analysis (TEA) and Life Cycle Assessment (LCA) to establish its industrial viability. The kinetic analysis revealed that SGSW decomposition is governed by a multi-stage mechanism with average activation energy. 50–70 kJ mol^−1^ across different models indicate favorable energy requirements for thermal conversion compared to several other biomass feedstocks. The Techno-Economic Analysis further validates the transition from lab-scale to industry, projecting a unit production cost of 1.05 USD per kg and a Net Present Value (NPV) of USD 6.67 million. Crucially, the process demonstrates a Return on Investment (ROI) of 38.87%, which significantly exceeds the standard strategic threshold of 20% for bioprocess ventures, alongside a rapid payback period of 2.57 years. TEA revealed that the unit production cost is approximately 1.05 USD per kg, and the payback time is around 2.57 years for a throughput of 0.33 T h^−1^ of SGSW. The LCA results underscores that SGSW biochar sustainability is sensitive to allocation methods, with GWP values ranging from 0.88 to 1.27 kg CO_2_ eq per kg of biochar. Conclusively, by establishing a robust link between favorable reaction kinetics, superior economic returns, and quantifiable environmental impacts, this work transforms SGSW from an underutilized agricultural residue into a strategic, data-backed feedstock for the global transition toward a circular bio-economy.

## Author contributions

Subramaniyasharma Sivaraman: writing – original draft, visualization, methodology, conceptualization, formal analysis, investigation. Rangabhashiyam Selvasembian: writing – review & editing, resources, supervision, conceptualization.

## Conflicts of interest

The authors declare that they have no known competing financial interests or personal relationships that could have appeared to influence the work reported in this paper.

## Data Availability

Data for this article are available at github at https://github.com/subramaniyasharma/SGSW-Pyrolysis.git.
